# Circulating Biomarkers for Early Stage Non-Small Cell Lung Carcinoma Detection: Supplementation to Low‐Dose Computed Tomography

**DOI:** 10.3389/fonc.2021.555331

**Published:** 2021-04-21

**Authors:** Chin Fung Kelvin Kan, Graham D. Unis, Luke Z. Li, Susan Gunn, Li Li, H. Peter Soyer, Mitchell S. Stark

**Affiliations:** ^1^ The University of Queensland, Ochsner Clinical School, Laboratory of Translational Cancer Research, Ochsner Clinic Foundation, New Orleans, LA, United States; ^2^ The University of Queensland Diamantina Institute, The University of Queensland, Dermatology Research Centre, Brisbane, QLD, Australia; ^3^ Department of General Surgery, Brigham and Women’s Hospital, Boston, MA, United States; ^4^ Department of Medicine, Ochsner Clinic Foundation, New Orleans, LA, United States; ^5^ Department of Medicine, Stamford Hospital, Columbia College of Physicians and Surgeons, Stamford, CT, United States; ^6^ Department of Pulmonary and Critical Care, Ochsner Clinic Foundation, New Orleans, LA, United States; ^7^ Department of Dermatology, Princess Alexandra Hospital, Brisbane, QLD, Australia

**Keywords:** microRNA, non-small cell lung cancer, biomarker, lung cancer, pulmonology, liquid biopsy

## Abstract

Lung cancer is currently the leading cause of cancer death in both developing and developed countries. Given that lung cancer has poor prognosis in later stages, it is essential to achieve an early diagnosis to maximize patients’ overall survival. Non-small cell lung cancer (NSCLC) is the most common form of primary lung cancer in both smokers and non-smokers. The current standard screening method, low‐dose computed tomography (LDCT), is the only radiological method that demonstrates to have mortality benefits across multiple large randomized clinical trials (RCT). However, these RCTs also found LDCT to have a significant false positive rate that results in unnecessary invasive biopsies being performed. Due to the lack of both sensitive and specific screening methods for the early detection of lung cancer, there is an urgent need for alternative minimally or non-invasive biomarkers that may provide diagnostic, and/or prognostic information. This has led to the identification of circulating biomarkers that can be readily detectable in blood and have been extensively studied as prognosis markers. Circulating microRNA (miRNA) in particular has been investigated for these purposes as an augmentation to LDCT, or as direct diagnosis of lung cancer. There is, however, a lack of consensus across the studies on which miRNAs are the most clinically useful. Besides miRNA, other potential circulating biomarkers include circulating tumor cells (CTCs), circulating tumor DNA (ctDNAs) and non-coding RNAs (ncRNAs). In this review, we provide the current outlook of several of these biomarkers for the early diagnosis of NSCLC.

## Introduction

Despite advances in detection and treatment for various types of cancer, lung cancer has remained one of the cancers with the highest incidence and mortality (1). In 2018, the World Health Organization (WHO) reported that lung cancer was the leading cancer diagnosed accounting for approximately 2.1 million cases in both developing and developed countries (1). Moreover, lung cancer has the highest cancer mortality, with 1.8 million deaths or 18.4% of all cancer deaths annually. Currently in the USA, the National Institutes of Health (NIH) data shows that 57% of all lung cancer is diagnosed at a distant organ metastasis, with an overall five-year survival of 19.4% (2). In fact, lung cancer causes more deaths than prostate, breast, colorectal and brain cancers combined (3). Given that lung cancer has such a poor survival profile, there is an urgent need to discover new treatment, and more importantly, sensitive and specific methods to detect lung cancer at an early stage to commence treatment as soon as possible.

Lung Cancer is divided into Small Cell Lung Cancer (SCLC) and Non-Small Cell Lung Cancer (NSCLC). Most cases are NSCLC (85%) compared with SCLC (15%) (4). NSCLC can be further divided into different subtypes. The most common subtypes include: Lung Adenocarcinoma (LUAD, 40%), squamous cell carcinoma (LUSC, 25%) and large cell carcinoma (10%) (5). Other less common NSCLC include neuroendocrine tumors and carcinomas with pleomorphic, sarcomatoid elements.

Low‐dose computed tomography (LDCT) is a radiological method that has been used to screen NSCLC. Once a suspicious nodule is identified using this method, an invasive biopsy will be performed. The National Lung Screening Trial (NLST), in 2010, has shown that LDCT decreased mortality by 20%, but alarmingly, this screening method had a 96.4% false positive rate (6), making it extremely non-specific and leading to many unnecessary invasive procedures and associated complications. This highlights the need for a NSCLC detection method that is both sensitive and specific.

Due to the lack of sensitive and/or specific radiological screening methods, there has been much research effort to investigate biomarkers to perform a “liquid biopsy.” Liquid biopsies are methods of screening or diagnosing diseases using saliva, urine, cerebrospinal, and blood (plasma and serum) biomarkers. Examples of these biomarkers include circulating tumor cells (CTCs), circulating tumor DNA (ctDNA), circulating microRNA (miRNA), non-coding RNA (ncRNAs), and tumor-derived extracellular vesicles (EVs) [reviewed in (7)]. Within this group, miRNAs have become one of the leading NSCLC biomarker candidates. MiRNAs are non-coding RNA which are ~22 nucleotides in length. Their primary function is to bind to messenger RNAs (mRNA) to restrict protein translation or increase mRNA degradation. In certain cancers, such as ovarian, lung or pancreatic cancers, changes in miRNA expression may provide both sensitive and specific diagnoses (8–10). More importantly, miRNA may be found in blood, stool, urine, and sputum, making their extraction minimal to non-invasive (11–15). Owing to these properties, miRNAs are also currently being investigated as biomarkers for disease prognosis and treatment response by surgical or medical therapy (16–18).

In this review, we provide a summary of the leading candidates for lung cancer liquid biopsies including miRNA, CTCs, ctDNA, and other ncRNAs. We highlight that when these biomarkers are used in conjunction with LDCT, it is possible to achieve both sensitive and specific early NSCLC detection.

## Current Radiological Screening Methods

The most common radiographical screening methods include using LDCT, and it is the only universally recommended method for lung cancer screening (19). The evidence to screen with LDCT is strong, and it is the only screening test shown to reduce the NSCLC mortality rate in multiple large randomized clinical trials (RCT) (6, 20, 21). The NLST screened 53,454 people aged between 55 to 74 years old with at least 30 pack-years, or have stopped smoking within the previous 15 years from 2002 to 2010 (6). The trial participants were screened once by LDCT when they were randomized, and then two additional times at yearly intervals (6). The study reported that there was actually a 20% reduction in mortality over a five-year period in the LDCT group as compared with the chest x-ray (CXR) group (6). Alarmingly, despite the high sensitivity of LDCT, the trial also discovered that LDCT had a 96% false-positive rate (6), making the test highly unspecific. Along with the US-based NLST trial, other European-based trials experienced similar results. The Nederlands-Leuvens Longkanker Screenings ONderzoek (NELSON) trial, the second-largest LDCT lung cancer screening RCT involving 15,792 individuals, showed that while there was no mortality benefit within five years of the annual LDCT screen, there was a 26% reduction in lung cancer death after 10 years of follow-up (20). The NELSON trial also had a high false positive rate (59.4%), highlighting that LDCT, when used in isolation, is highly non-specific (20). Smaller European trials, such as the Multicentric Italian Lung Detection (MILD) trial, and the Danish Lung Cancer Screening Trial (DLCST), also showed a LDCT false positive rate ranging from 18.5% to 67.5% (21, 22).

Such high false-positive rates, and over diagnosis, significantly increase the use of unnecessary invasive procedures and may lead to complications. Furthermore, annual LDCT screening also leads to increased radiation exposure. For example, in the NLST, an average radiation exposure of 8 mSv over three years could potentially cause one cancer in every 2500 people screened (23). Therefore, there have been efforts to combine biomarkers with imaging to permit a decrease in radiation exposure. Using 939 study participants from the MILD study (10-year follow-up RCT screening 4,099 participants aged 49-75 years with a smoking history of more than 20 years), Sozzi et al. (24) screened a previously discovered panel of 24 miRNAs (25). This retrospective analysis of a large patient cohort revealed that the miRNA panel could reduce LDCT false positives from 19.6% to only 3.7% (24). Due to the success of this retrospective analysis, a prospective clinical trial is currently on-going (BioMILD) (26).

### Potential Screening Methods by Liquid Biopsy

To reduce unnecessary invasive procedures and radiation exposure, and increased screening coverage for lung cancer, there is an urgent need for a cheap, sensitive and specific screening test that could be used in conjunction with LDCT. The “liquid biopsy” field is rapidly expanding in the field of translational cancer research (27). Biomarkers such as circulating tumor DNA (ctDNA), circulating tumor cells (CTC), miRNA, circulating ncRNA, and many others have been explored as potential clinical markers for detection and prognosis in different cancers such as prostate, lung, and melanoma (27). Out of all the biomarkers, the use of miRNAs has the greatest potential, due to its simplified extraction methods (from blood, saliva, and urine), sensitivity, and well-established role in messenger RNA (mRNA) regulation. In fact, having a liquid biopsy as a molecular test for patients with NSCLC, is included amongst a list of recommendations in the new guidelines for the College of American Pathologists (CAP), as well as the International Association for the Study of Lung Cancer (IASLC), and the Association for Molecular Pathology (AMP) (28).

There are many reasons why a liquid biopsy is useful: Firstly, a significant patient subgroup cannot undergo conventional biopsy procedures or be induced under anesthesia for surgery due to poor clinical condition or the location of the tumor. Secondly, liquid biopsies spare the patient from surgery complications from CT-guided transthoracic lung biopsies. Thirdly, in a single tissue biopsy, the amount of tissue may not be enough to perform all the required tests or analyses, which is compounded by the heterogeneous nature of the tumor. Finally, invasive procedures or surgeries are more expensive than a blood draw, making non-invasive markers more cost-effective (28).

## Potential NSCLC Biomarkers

There are a number of potential biomarkers currently being investigated or studied. In the next sections, we will discuss such biomarkers’ utility, current status, as well as pros and cons. A summary of the various biomarkers and associated clinical trials are shown in **Tables 1**–**3**.

**Table 1:** Circulating biomarker candidates for NSCLC diagnosis.

Biomarker class	Description	Detection method	Commercial product(s)
Circulating Tumor Cells (CTCs)	Individual cells detached from the tumors and circulate in the body, which can be detected mainly in whole blood but can also be found in other body fluids such as pleural effusion	ISET™, CellSearch™, CTC-iChipneg device, ClearCell FX™, CellSieve™, Parsortix™ ISET, GILUPI CellCollector™ (29)	CellSearch™
Circulating Tumor DNA (CtDNA)	Circulating free tumor DNA is single or double-stranded DNA from CTCs or the tumor itself	ME-PCR, ARMS, BEAMing, Sequenom, AS-APEX, ARMS, cpbas EGFR blood test, PNA-mediated PCT clamping, MBP-QP, ddPCR (30)	cobas™ EGFR Mutation Test v2, Guardant360™, InVisionFirst™-Lung

**Table 2:** Ongoing clinical trials studying using microRNA to detect NSCLC.

Trial number	Sponsoring institution	Trial name	Outcome measures	Reference
NCT02247453	Ugo Pastorino, Fondazione IRCCS Istituto Nazionale dei Tumori, Milano	Plasma microRNA Profiling as First Line Screening Test for Lung Cancer Detection: a Prospective Study (BioMILD)	24 miRNA panel as adjunct diagnosis tool with LDCT	(52)
NCT03452514	Hummingbird Diagnostics	Addition of microRNA Blood Test to Lung Cancer Screening Low Dose CT	Validation study for circulating miRNA to be used with LDCT. Did not publish miRNA panel.	(53)
NCT04323579	Istituto Clinico Humanitas	Validation of Multiparametric Models and Circulating and Imaging Biomarkers to Improve Lung Cancer EARLY Detection. (CLEARLY)	Diagnose NSCLC with 45- and 16-miRNA signatures combined with LDCT	(54)
NCT03397355	China-Japan Friendship Hospital	Monitoring the Changes of Tumor-related Biomarkers Before and After Pulmonary Nodule Biopsy.	Identify circulating miRNA changes before and after tumor resection	(55)
NCT03721120	Centre Leon Berard	Evaluation of the Feasibility and Clinical Relevance of Liquid Biopsy in Patients With Suspicious Metastatic Lung Cancer (LIBELULE)	Circulating miRNA profiling before treatment and 8 weeks after treatment initiation	(56)

**Table 3:** Circulating ncRNA candidates for NSCLC diagnosis.

NcRNA	Description	Clinical trials
tRNA-derived small RNA (tsRNA)	NcRNAs generated from precursor or mature tRNAs	Not in clinical trial
Piwi-interacting RNA (piRNA)	NcRNA that are 23 to 32 nt in length and generated through a non-Dicer pathway from a single stranded RNA	Not in clinical trial
Long non-coding RNA (lncRNA)	More than 200 nt and does not have an open reading frame (ORF)	NCT03830619
Pseudogenes	NcRNA that have nucleotide sequence that is similar to another gene but defective	Not in clinical trial
Circular RNA (circRNA)	NcRNA with closed loops instead of being a linear form. Known to sequestering miRNA.	Not in clinical trial

### MicroRNA (miRNA)

MiRNA dysregulation is well known to be associated with a wide range of diseases, including cancer, due to their important function in gene regulation. It has been long known that miRNA dysregulation can lead to certain oncogene upregulation or tumor suppressor gene down-regulation (31). This dysregulation has been shown to down-regulate protective genes such as *PTEN* and *TP53* (32, 33) while increasing the expression of oncogene such as *RAS* and *MYC* (34, 35). For example, cigarette smoke has been shown to downregulate the miRNA let-7c, a known regulator of the oncogene *KRAS*, which is known to induce tumorigenesis (36). This suggests that dysregulation of miRNA may be an early event in lung cancer tumorigenesis and may contribute to cancer progression (37). It can be postulated that by detecting such changes may also contribute to the early detection of cancer.

There are several reasons that make miRNA attractive as a detection tool. Primarily, it can be sensitively detected, and extracted non-invasively (38). Additionally, miRNA has been known to be very stable in bodily fluids, including serum and plasma, thus making it a readily obtainable biomarker (38). Interestingly, in different lung cancer subtypes such as squamous cell carcinoma, adenocarcinomas, and small cell carcinoma, miRNA expression can behave in a tissue-specific manner (39, 40).

In general, miRNAs are released from cells *via* distinct exosomal or non-exosomal pathways. The three main theories on miRNA secretion into blood are: 1) Leakage due to chronic inflammation and/or cell apoptosis 2) Active loading into exosomes, which are 40-100nm vesicles, released from the tumor cell and 3) Active secretion and bound to proteins (e.g. AGO2) or lipoproteins (LDL, HDL) (41, 42).

Given that there is dysregulation of miRNA in tumor cells, in theory there should be changes in multiple miRNA levels, which could be detected in body fluids, including plasma, whole blood, sputum, and urine (38). For example, a study by Yanaihara et al. showed that 12 miRNAs could be used to discriminate NSCLC from non-cancerous lung tissues (43). Whereas a review by Moretti et al. identified, through literature search, one highly specific and one highly sensitive miRNA panel respectively to detect stage I and II NSCLC. This review proposed that a two-phase screening may be appropriate, where the highly sensitive panel is used first, and then the highly specific panel can confirm the finding of the first panel, and by doing so, this could increase the overall detection sensitivity to 91.6%, with an overall specificity of 93.4% (44).

As described, miRNAs can also be used to distinguish subtypes of lung cancer. For example, miR-7c, miR-21, miR-29b, miR-106a, miR-125a-5p, miR-129-3p, miR-205, and miR-375 can be used to distinguish the subtypes of NSCLC and SCLC with an accuracy of 93.7% (45). While there are yet to be large scale validation studies of NSCLC miRNA detection panels, there have been promising results in other cancer types. Recently, Ochiya et al. have shown that a panel miRNA can be used to detect early stages of ovarian cancer with 99% sensitivity and 100% specificity (39). Therefore, there is much potential for the discovery of a highly sensitive and specific NSCLC miRNA panel to to improve patient outcomes by detecting disease in its earliest stage so treatment can commence without delay.

Besides endogenous gene regulation, miRNA can be transmitted in the blood and act in cell-to-cell communication similar to the mode of action of a hormone (46). This is interesting as it suggests that certain “hormone-like” miRNA expression may change in the blood of NSCLC patients, thus supporting the hypothesis that changes in plasma miRNA could be used to detect the development and the spread of cancer. For example, miR-233 has been identified as a “hormone-like” miRNA, and has been reported to transport into breast cancer cell with no cell-to-cell contact (47). This further solidifies that miRNA expression change may be early events of carcinogenesis and that miRNA detection can provide early indications of tumorigenesis and metastasis.

Due to cancer’s diverse nature, it can be very difficult to accurately predict the prognosis of patients, as it can be influenced by lifestyle and specific driver mutations unique to the cancers. Research performed in metastatic renal cell carcinoma showed that stem cells release microvesicles that carry several miRNAs that were associated with the establishment of a metastatic friendly environment in the lung (48). In terms of NSCLC, high expression of tissue let-7a, miR-221, miR-137, and miR-182 have been shown to increase the invasiveness of lung cancer cells and predict poor prognosis (49). While Kim et al. has shown that miR-126 reduction and miR-200c elevations in biopsied samples indicate poor prognosis in NSCLC patients (50). Finally, a meta-analysis of miRNAs by Yu et al. demonstrated that a global increase in overall miRNA level in NSCLC patients tended to be associated with decreased survival (51).

Plasma-derived expression of miRNAs for NSCLC detection, is a minimally invasive procedure, currently at an early phase of development. There are a number of current clinical trials using miRNAs for NSCLC detection (summarized in **Table 2**). Sozzi et al. showed that a panel of 24 plasma miRNA have 87% sensitivity and 81% specificity, for detecting lung cancer, and have a 27% positive predictive value, and 99% negative predictive value (24). This miRNA panel is currently undergoing the BioMILD trial (**Table 2**), which may provide evidence on the utility of miRNA alongside LDCT to diagnosis NSCLC. Preliminary data from the BioMILD trial panel suggest that it may be able to screen for patients who are high risk in lung cancer development. Furthermore, the Sozzi et al. study was able to identify patients who were at high risk of developing lung cancer up to two years before radiological diagnosis (57).

Rosetta Genomics (not active at time of review) was approved by the Food Administration Agency (FDA) in 2012, to use a library of miRNA to differentiate between small cell lung cancer, carcinoid, squamous non-small cell lung cancer, and non-squamous cell non-small cell lung cancer (40). However, this miRNA panel was only to be used on tissue biopsy samples, which restricted its use as early detection of lung cancer and as a minimally invasive procedure (58). As such, identifying miRNA in plasma, which can detect different subtypes of NSCLC, can be a future path when investigating the relationship of miRNA and NSCLC.

Hummingbird diagnostics is currently developing NSCLC, melanoma, breast cancer, and general cancer miRNA panels (59). Their 17 patents granted in the field of whole blood miRNA expression profiling are currently being clinically validated with funding from three European FP-7 consortia (BestAgeing, RiskyCAD, and EURenOmics) (59). Due to this early phase, it has much potential, and can hopefully be translated into clinical use in the future. As such, identifying miRNA in plasma, which can detect different subtypes of NSCLC, has promise in the minimally invasive diagnosis of NSCLC. There has been a lack of investigations to study miRNA panels intended to separate early-stage squamous cell carcinoma and adenocarcinoma, the two most common NSCLC subtypes.

### Circulating Tumor Cells (CTCs)

Circulating Tumor Cells (CTCs) have been a popular research topic for cancer detection in recent years. CTCs are cells detached from tumors, which can be detected mainly in whole blood, but can also be found in other body fluids such as pleural effusions (60). Since CTCs can represent the malignancy, it has the potential to be used as a minimally invasive biomarker to understand the tumor’s biology and associated somatic mutations and copy number aberrations. Furthermore, it has been reported that CTCs have an important role in cancer metastasis as they will invade venous or lymphatic vessels, and seed in different organs; thus CTCs may be a useful tool in the detection of metastatic cancer (61). As such, there are several phase II and III clinical trials undergoing investigation for the number of CTCs in blood and their relationship with treatment response. For example, in the Treat-CTC (NCT01548677) Phase II RCT, the investigators are investigating the correlation between CTCs and disease survival in patients with human epidermal growth factor receptor 2 (HER2) negative primary breast cancer treated with trastuzumab therapy.

Besides using the amount of CTCs in the blood as an overall detection for metastatic cancer, it has been reported that protein, and mRNA from the CTCs can be used to determine specific mutant protein and prognosis (62). For example, the presence of *EpCAM/MUC1* mRNA-positive CTCs significantly decrease a NSCLC patient’s disease-free, and overall survival (63). Furthermore, high amounts of CTCs collected from the pulmonary vein (≥18 PV-CTCs/7.5ml), has been found to be associated with decreases in survival of early staged NSCLC patients (64). Interestingly, the commercially available nanoString nCounter platforms have been shown to be able to measure plasma CTC-RNAs. This platform can directly measure mRNA without requiring its conversion to cDNA or multiplexed target enrichment (MTE), by directly capturing and counting of individual targets (65). In fact, the FDA approved the nCounter-based Prosigna assay in 2013 to diagnosis breast cancer subtypes, and risk of recurrence, by using a 50-gene signature in breast tissue samples (66). The nCounter system also has been shown to be able to detect EV-derived mRNA (67). Thus, there is potential to use this platform to detect gene expression in CTCs with high sensitivity.

CTCs can also be analyzed to detect common somatic *EGFR* mutations (68) as well as *ALK* translocations using filter-adapted fluorescent *in situ* hybridization (FA-FISH) (69). As mentioned above, since NSCLC treatments may involve targeting specific mutations, requiring fine-needle biopsy of the tumor for mutation analysis, CTC mutation analysis may provide a non-invasive method of determining a tumors mutation. The detection of CTCs is however limited to only a few tumor cells per 1x10^6^-10^7^ peripheral blood mononuclear cells in patients with advanced tumors (27). The Treat-CTC study also reported that most patients only have one CTCs per 15 ml of blood, thus making it a major limitation to the study (70) using current detection methods. Since CTCs are present in low numbers in blood, detection requires highly sensitive and specific methods. The current FDA approved CellSearch^®^ (Veridex LLC) uses EpCAM-coated magnetic beads to isolate CTCs; it is currently approved for metastatic breast, colorectal, and prostate cancers (71). There have been studies investigating CellSearch™ as a tool for NSCLC, and it has been reported that in metastatic NSCLC, only 32% of patients have more than two CTCs before chemotherapy treatment (27). Whilst high specificity of more than 89% can be achieved, sensitivity varies from 27-70% (27), which may limit its clinical scope in NSCLC diagnoses. Even though this method is FDA approved, and that detection sensitivity of CTC in late-stage NSCLC is improved significantly (27), this method has little clinical utility for early detection of NSCLC.

Other more recent CTC isolation systems, such as ClearCell^®^ FX (72), the VTX-1 Liquid Biopsy System (73), and the Parsortix™ Cell Separation System (74), have been shown to improve CTC capture to the point where there are enough tumor cells to be xenografted into mice, and to study the tumor cells’ molecular biology (75, 76). However, these studies mainly focus on SCLC as these tumors have high numbers of CTCs in blood, with a strong ability to proliferate (75). In comparison, NSCLC have fewer CTCs in the blood, and lower relative proliferative abilities, especially during early cancer stages. This makes NSCLC CTCs much harder to capture and study (75).

### Circulating Free Tumor DNA (ctDNA)

Circulating free tumor DNA is single or double-stranded DNA from CTCs or the tumor itself. CtDNA exists in serum or plasma and may preserve many mutation characteristics of the tumor cells (77). For example, it can preserve somatic point mutations or methylation patterns of metastatic colorectal cancer cells with *KRAS* mutation, which have been used to predict the patient’s prognosis (78). Moreover, ctDNA has been reported to correlate with tumor burden and to the therapeutic response in NSCLC. A meta-analysis by Fan et al. showed that in groups that were treated with or without chemotherapy, detection of the *EGFR* mutation in ctDNA improved patient prognosis, while patients with the *KRAS* mutation had a worse prognosis in NSCLC (79).

Since many cancer treatments target specific mutations, and cancer can develop drug resistance quickly, it is important to rapidly determine a tumor’s mutation status. While invasive tumor biopsy and immunohistochemical staining are the gold standards in mutation determination (80), ctDNA may be representative of the genome of the tumor. Thus, ctDNA can be used to identify mutations non-invasively (80, 81). In fact, the cobas^®^ EGFR Mutation Test v2 from Roche was FDA approved to detect NSCLC *EGFR* exon 19 or exon 21 substitution mutations, in 2016 (82). Indeed, in more recent large clinical trials, (e.g. AURA2, AURA3 and FLAURA), it has been demonstrated that it is possible to use cobas^®^ to detect ctDNA with EGFR mutations in locally advanced and metastatic NSCLC. For example, the AURA3 trial used ctDNA to gauge NSCLC response to osimertinib as compared with platinum-based therapy plus pemetrexed. This study supported that ctDNA can be used to detect EGFR T790M mutations in plasma, though the study also cited that there can be a high false negative rate (83). Similarly, the AURA2 trial also exhibited low sensitivity, with circulating T790M mutation only present in 40% of patients. Thus, it was suggested that patients who were plasma ctDNA negative should obtain a tissue DNA sample for verification (84). The FLAURA trial, also investigated circulating levels of T790M mutations and found that detectable signals were higher in patients that had a higher tumor burden due to increased shedding of ctDNA. Furthermore, this study reported that there were patients with “non-shedding” tumors with no detectable plasma ctDNA and these patients had better prognosis than patients with detectable plasma ctDNA (85). Thus, plasma ctDNA may be specific in detecting later staged NSCLC, and their mutation status, but it may not be sensitive enough to detect early staged NSCLC.

Other clinical trials also found that while ctDNA is highly specific, it may not be as sensitive as miRNA, with early trials ranging between 43-65.7% in detecting malignancy mutations (28, 86). For example, the IPASS and LUX-LUNG3 Phase III RCTs showed that sensitivity level was only 43% (28). Whereas the ASSESS trial showed that ctDNA can detect EGFR mutations with low sensitivity (46%), but a high specificity (97%) (28). Later trials, such as the phase IV IFUM trial, have found that the sensitivity to detect ctDNA was improved up to 65.7% using the Scorpion Amplification Refractory Mutation System (ARMS)-based EGFR mutation detection kit (86). Interestingly, different subtypes of lung cancer may secrete a different amount of ctDNA due to the degree of necrosis in early staged NSCLC. In a study by Abbosh et al., a commercial ctDNA assay panel (developed by Natera) was used to screen the first 100 stage I (LUSC and LUAD) TRACERx study participants; which resulted in ctDNA being detected in 94% of LUSC, whereas only 13% of LUAD patients were positive. (87). Since LUAD is the largest subtype of NSCLC and any lung cancer (5), the use of ctDNA is limited to only late stage, and thus need to be combined with other biomarkers or imaging modalities to detect early stage NSCLC.

There are multiple ways of detecting ctDNA, which includes PCR based methods and Next-Generation Sequencing (NGS) based methods. PCR based method examples include quantitative PCR (qPCR), digital PCR (dPCR), and BEAMing (which stands for beads, emulsion, amplification and magnetics) (27). In contrast, NGS based methods include CAncer Personalized Profiling by deep Sequencing (CAPP-Seq), and Tagged AMplicon deep Sequencing (TAM-Seq) (88). Compared to PCR based methods, NGS based methods are more expensive, but their sensitivity and specificity are very promising. CAPP-seq has been assessed to have an initial sensitivity and specificity of 85% and 96% (88). However when stage 1 tumors where specifically assessed, the sensitivity was reduced to 50% (88). A recent paper by Chabon et al. showed that by optimizing the CAPP-seq methodology, and using machine learning to generate a lung cancer likelihood in plasma score (Lung-CLiP), stage 1 NSCLC can be detected with 41% sensitivity and 98% specificity, while stage 2 can be detected with 54% sensitivity (89). Moreover, as oppose to PCR based methods, NGS uses an unbiased approach, assessing all genes, and as such can detect unknown gene mutations of lung cancer.

DNA methylation dysregulation is a hallmark of carcinogenesis, as CpG island hypermethylation can reduce the expression of certain onco-protection genes. Since ctDNA is mainly derived from apoptotic or dead tumor cells, they should represent the tumor genomic DNA (90). In fact, Hao et al. have shown that methylated ctDNA was identified in 32 of 34 (94%) colo-rectal cancers which had metastasized to the lung (91). This indicates that ctDNA may be able to help identify if the lung tumor is of primary, or metastatic origin. Currently, the MEDAL trial is the first prospective study to compare the detection of aberrant methylation and mutations in ctDNA among stage 1A to 3 surgical NSCLC patients (92). The MEDAL trial results are due in 2021, and it will be interesting to see if they provide evidence to support using methylated ctDNA for NSCLC detection.

There have been many questions about the use of ctDNA as an early diagnosis tool. Like CTCs, ctDNA has been reported to have very low amount in blood ranging normally from 13-51 ng/ml (93). This requires large volumes of blood to be drawn or high levels of secretion. Moreover, the cancer may need to be relatively advanced before sufficient ctDNA can be extracted to cross the detection threshold, thus making it more useful to detect later stages of the cancer. Finally, while cobas^®^ can reach acceptable levels of specificity, sensitivity issues remain problematic. Since detection of tumor specific mutations are possible, this high specificity may make the assessment of ctDNA an ideal candidate to be used in conjunction with LDCT. Indeed, the CancerSEEK trial showed that ctDNA has a median of 70% sensitivity across eight cancer types, when combined with protein biomarkers, in 1,015 patient samples, which included lung cancer (94).

GRAIL Inc is currently conducting four large scale ctDNA randomized control trials (RCT): the SUMMIT trial (95), the PATHFINDER trial (96), the Circulating Cell-free Genome Atlas (CCGA) Study (97) and the STRIVE study (98). Out of the four RCTs, the SUMMIT trial specifically aims to evaluate the performance of the GRAIL ultra-deep sequencing to detect ctDNA in patients at high-risk for developing lung cancer, with the levels of ctDNA detected correlated with LDCT outcomes (95). The trial will enroll 25,000 participants deemed at high risk, i.e., at least 30 pack years of smoking, and if a former smoker, have quit in the past 15 years: or having a Prostate, Lung, Colorectal and Ovarian (PLCOm2012) risk score ≥1.3% (6-year lung cancer risk).

### Non-coding RNA (ncRNA)

Non-coding RNA (ncRNA) is an emerging field in disease treatment and detection, which has been gaining traction in the last few years. For a long time, researchers considered ncRNA as background noise, however recent discoveries have shown that ncRNA have a significant role in malignancy development. Currently, there are six main ncRNA classes: MicroRNA (miRNA), transfer RNA-derived small RNAs (tsRNAs), PIWI-interacting RNA (piRNAs), long ncRNAs (lnRNAs), pseudogenes and circular RNAs (circRNAs) (99). A summary of these ncRNAs that are potential biomarkers for NSCLC is summarized in **Table 3**.

TsRNAs are a group of ncRNAs discovered in the last decade. They are modified transfer RNAs altered by a still unknown enzyme, and have been found to regulate mRNA by binding to its 3’UTR, 5’UTR or the reading frame (99). There are reports that certain tsRNAs are significantly upregulated in different types of cancer. For instance, ts-46 and ts-47 are associated with both chronic lymphocytic leukemia and lung cancer (100). In fact, the over-expression of both ts-46 and ts-47 has been shown to reduce lung cancer cell line proliferation (100). Due to the changes in expression during the diseased state, tsRNA has been suggested to be used as a minimally invasive cancer biomarker. In a study by Zhu et al., tRNA-ValTAC-3, tRNA-GlyTCC-5, tRNA-ValAAC-5, and tRNA-GluCTC-5 were increased significantly in plasma-derived exosomes from liver cancer patients (101). Since very few studies have been performed on tsRNAs, their precise role in cell biology remains relatively unknown. Therefore, more studies are essential before its clinical utility can be determined.

PiRNAs are another group of newly discovered ncRNAs. They are 24-31 nt in length (102, 103), and are loaded into the PIWI family of Argonaut proteins, which then silence transposons in the gonads (99). Since they are mainly expressed in gonadal cells, under normal circumstances, they are expressed at low levels in somatic tissues (99, 104). There have been reports that cancer and healthy blood and tissues have distinct piRNA signals (105). For example, piR-823 is highly expressed in serum and urine of a patient with advanced renal cell carcinoma (103). Like tsRNAs, because of this change in expression, reports suggest that piRNA may be used as either a prognostic or diagnostic biomarker. For example, the expression of piR-1245, from colorectal carcinoma tissue, has been shown as an independent prognostic biomarker (106). Interestingly, piRNAs are similar in size and stability with miRNA, though it is much less well-studied (99). Due to its incomplete biological understanding, and immature detection methods, more studies must focus on piRNA before it can become a biomarker candidate for disease diagnosis.

LncRNA is an emerging field in disease treatment and detection. Recent research has suggested that lncRNA regulate the development and apoptosis of cells through various mechanisms, such as; acting as a decoy, as a signaling molecule, as a guiding transcription factor, or as a scaffold for genomic DNA histones (107). Similar to miRNA, lncRNA are relatively stable in body fluids and seem to be tissue-specific (108). In contrast to miRNA, which can be exocytosed, lncRNAs are released into the blood when tumor cells undergoes apoptosis or lysis (108). Similar to miRNA, lncRNA can be potentially used to detect pathological conditions. For example, detection of *PCAT2* has been shown to be a more sensitive and specific marker for prostate cancer than prostate-specific antigen (PSA) (109). While in NSCLC, *MALAT-1* has been shown to have a sensitivity of 56% and specificity of 96% for detection (110). LncRNA have complex direct and indirect regulation and multifocal effects on both mRNA transcription and protein translation. Thus, more research will be needed before lncRNA can be used in disease detection and treatment. Currently, several clinical trials are studying the use of lncRNA as a prognostic or detection biomarker. Two such clinical trials are underway, NCT02641847 which studies using lncRNA to measure triple-negative breast cancer response, and NCT03057171 is studying the use of lncRNA to detect MALT lymphoma in the stomach.

Pseudogenes are a subclass of lncRNA transcripts. Their nucleotide sequence can be very similar to their corresponding mRNA transcript, which allows miRNAs to bind to it, thus acting as a miRNA decoy (99). For example, *BRAFP1* pseudogene acts as a miRNA decoy for *BRAF*, thus leading to an increase in BRAF expression and increased cell proliferation (111). Due to its relevance in protein expression, there have been efforts to use it as a diagnostic marker. Kalyana-Sundaram et al. identified 218 pseudogenes expressed only in cancer but not in healthy individuals (112). This makes it a strong target for the development of a multi-panel cancer screening tool. Another study, in gastric cancer, showed that there were lower levels of the pseudogene *PTENP1* compared to healthy individuals, which when used along with the expression level of two lnRNAs (*CUDR* and *LSINCT-5*), have high diagnostic power (113). Pseudogenes are also being investigated as a prognostic biomarker. Two distinct clusters of 241 and 205 pseudogenes may be able to predict survival time in kidney cancer (114). Even though there are some studies suggesting that pseudogenes may have clinical significance, it is much less studied than lncRNA. Like many ncRNAs, more studies need to be performed before they can become reliable diagnostic and prognostic biomarkers.

CircRNAs are a group of endogenous noncoding RNA with closed loops instead of the traditional linear form (115). They seem to closely regulate miRNA expression and function, thus indirectly affecting miRNA downstream pathways (107). For example, ciRS-7 contains 70 selectively conserved miRNA binding sites and have been shown to be associated with Argonaute (AGO protein) to act as a miRNA sponge for miR-138 (116). It has also been reported that in esophageal squamous cell carcinoma, that the circRNA, cir-ITCH, serves as a sponge for miR-7, miR-17, and miR-214 (117). Given that in different cancers, there can be upregulation or downregulation of circRNA ranging from two to six-fold (118), this dysregulation can contribute to the dysregulation of miRNA by upstream regulation.

## Conclusion and Future Directions

NSCLC remains one of the cancers with the highest incidence and mortalities (119). Many of those diagnosed, present with advanced or metastatic tumors, and therefore there is an urgent need for a screening test for early NSCLC diagnosis and treatment to increase survival (120). Liquid biopsy has become an attractive screening method due to its minimally invasiveness and sensitivity. However, there are many biomarker candidates currently being developed. In this review, we have discussed the use of miRNA as a NSCLC screening tool, as well as CTCs, ctDNAs and other ncRNAs.

**Figure 1:**
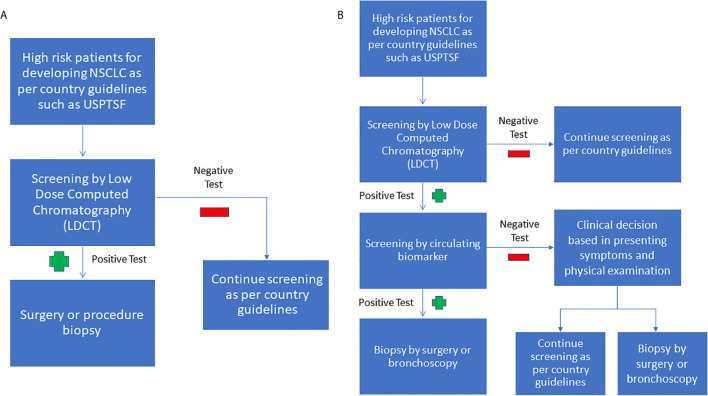
**(A)** Current NSCLC screening flowchart **(B)** Purposed combined screening radiologic imaging with biomarkers. As LDCT is sensitive but unspecific, it can be used as an initial screening test. If the LDCT is positive, the next step we purposed is to use circulating biomarkers to increase the LDCT’s specificity. If the LDCT is positive, but the biomarker screen is negative, we purpose to base the decision to perform a tissue biopsy on the clinician’s judgment.

Despite the significant effort to identify and develop standalone, minimally invasive biomarkers panels for NSCLC, a more likely clinically scenario will be the use of a combination of biomarkers and radiological evidence to aid in early diagnosis. We have summarized this approach in **Figure 1** and purposed to continue using LDCT to screen for NSCLC in high risk population due to its sensitivity. Furthermore, multiple clinical trials have shown that LDCT may reduce cancer mortality in the long run. If the LDCT is positive, a circulating biomarker test can be used to increase the specificity and to reduce the false-positive of LDCT. If the LDCT is positive, but the circulating biomarker test is negative, the clinician can decide if the patient requires a tissue biopsy, or can return for a LDCT screening test later, based on the countries’ guidelines, presenting symptoms, and physical examination.

## Author Contributions

CFKK, SG, LL, HPS, and MSS conceived the overall topics of discussion. CFKK and MSS drafted the original manuscript. CFKK, GDU, LZL, and MSS made substantial edits to the revised manuscript. All authors contributed to the article and approved the submitted version.

## Funding

This work was funded by the Merchant Charitable Foundation. CFKK was supported by The University of Queensland Research Training Tuition Fee Offset and The University of Queensland Research Training Living Stipend. MSS holds a National Health and Medical Research Council Fellowship (APP1106491), and HPS holds a National Health and Medical Research Council Medical Research Future Fund Next Generation Clinical Researchers Program Practitioner Fellowship (APP1137127).

## Conflict of Interest

The authors declare that the research was conducted in the absence of any commercial or financial relationships that could be construed as a potential conflict of interest.

## References

[1] Bray F Ferlay J Soerjomataram I Siegel RL Torre LA Jemal A. Global cancer statistics 2018: GLOBOCAN estimates of incidence and mortality worldwide for 36 cancers in 185 countries. CA Cancer J Clin (2018) 68(6):394–424. 10.3322/caac.21492 30207593

[2] Howlader N NA Krapcho M Miller D Brest A Yu M Ruhl J. SEER Cancer Statistics Review, 1975-2017. USA: National Cancer Institute Bethesda, MD (2020). Available at: https://seercancergov/csr/1975_2017/. based on November 2019 SEER data submission, posted to the SEER web site.

[3] Siegel RL Miller KD Jemal A. Cancer statistics, 2020. CA Cancer J Clin (2020) 70(1):7–30. 10.3322/caac.21590 31912902

[4] Molina JR Yang P Cassivi SD Schild SE Adjei AA. Non-small cell lung cancer: epidemiology, risk factors, treatment, and survivorship. Mayo Clin Proc (2008) 83(5):584–94. 10.4065/83.5.584 PMC2718421 18452692

[5] Zappa C Mousa SA. Non-small cell lung cancer: current treatment and future advances. Transl Lung Cancer Res (2016) 5(3):288–300. 10.21037/tlcr.2016.06.07 27413711 PMC4931124

[6] National Lung Screening Trial Research T Aberle DR Adams AM Berg CD Black WC Clapp JD. Reduced lung-cancer mortality with low-dose computed tomographic screening. N Engl J Med (2011) 365(5):395–409. 10.1056/NEJMoa1102873 21714641 PMC4356534

[7] Lianidou E Pantel K. Liquid biopsies. Genes Chromosomes Cancer (2019) 58(4):219–32. 10.1002/gcc.22695 30382599

[8] Wang W Wu LR Li C Zhou X Liu P Jia X. Five serum microRNAs for detection and predicting of ovarian cancer. Eur J Obstetrics Gynecol Reprod Biol: X (2019) 3:100017. 10.1016/j.eurox.2019.100017 PMC6687444 31404211

[9] Abreu FB Liu X Tsongalis GJ. miRNA analysis in pancreatic cancer: the Dartmouth experience. Clin Chem Lab Med (2017) 55(5):755–62. 10.1515/cclm-2017-0046 28343174

[10] Inamura K Ishikawa Y. MicroRNA In Lung Cancer: Novel Biomarkers and Potential Tools for Treatment. J Clin Med (2016) 5(3):36. 10.3390/jcm5030036 PMC4810107 27005669

[11] Wang K Yuan Y Cho JH McClarty S Baxter D Galas DJ. Comparing the MicroRNA spectrum between serum and plasma. PloS One (2012) 7(7):e41561. 10.1371/journal.pone.0041561 22859996 PMC3409228

[12] Pritchard CC Kroh E Wood B Arroyo JD Dougherty KJ Miyaji MM. Blood cell origin of circulating microRNAs: a cautionary note for cancer biomarker studies. Cancer Prev Res (Phila) (2012) 5(3):492–7. 10.1158/1940-6207.CAPR-11-0370 PMC4186243 22158052

[13] Link A Balaguer F Shen Y Nagasaka T Lozano JJ Boland CR. Fecal MicroRNAs as novel biomarkers for colon cancer screening. Cancer Epidemiol Biomarkers Prev (2010) 19(7):1766–74. 10.1158/1055-9965.EPI-10-0027 PMC2901410 20551304

[14] Mall C Rocke DM Durbin-Johnson B Weiss RH. Stability of miRNA in human urine supports its biomarker potential. Biomark Med (2013) 7(4):623–31. 10.2217/bmm.13.44 PMC3885156 23905899

[15] Xing L Su J Guarnera MA Zhang H Cai L Zhou R. Sputum microRNA biomarkers for identifying lung cancer in indeterminate solitary pulmonary nodules. Clin Cancer Res (2015) 21(2):484–9. 10.1158/1078-0432.CCR-14-1873 PMC4299920 25593345

[16] Xiao W Zhong Y Wu L Yang D Ye S Zhang M. Prognostic value of microRNAs in lung cancer: A systematic review and meta-analysis. Mol Clin Oncol (2019) 10(1):67–77. 10.3892/mco.2018.1763 30655979 PMC6313946

[17] Hojbjerg JA Ebert EBF Clement MS Winther-Larsen A Meldgaard P Sorensen B. Circulating miR-30b and miR-30c predict erlotinib response in EGFR-mutated non-small cell lung cancer patients. Lung Cancer (2019) 135:92–6. 10.1016/j.lungcan.2019.07.005 31447008

[18] Filipow S Laczmanski L. Blood Circulating miRNAs as Cancer Biomarkers for Diagnosis and Surgical Treatment Response. Front Genet (2019) 10:169. 10.3389/fgene.2019.00169 30915102 PMC6421259

[19] Mazzone PJ Silvestri GA Patel S Kanne JP Kinsinger LS Wiener RS. Screening for Lung Cancer: CHEST Guideline and Expert Panel Report. Chest (2018) 153(4):954–85. 10.1016/j.chest.2018.01.016 29374513

[20] de Koning HJ van der Aalst CM de Jong PA Scholten ET Nackaerts K Heuvelmans MA. Reduced Lung-Cancer Mortality with Volume CT Screening in a Randomized Trial. N Engl J Med (2020) 382(6):503–13. 10.1056/NEJMoa1911793 31995683

[21] Pastorino U Silva M Sestini S Sabia F Boeri M Cantarutti A. Prolonged lung cancer screening reduced 10-year mortality in the MILD trial: new confirmation of lung cancer screening efficacy. Ann Oncol (2019) 30(7):1162–9. 10.1093/annonc/mdz117 PMC6637372 30937431

[22] Pedersen JH Ashraf H Dirksen A Bach K Hansen H Toennesen P. The Danish randomized lung cancer CT screening trial–overall design and results of the prevalence round. J Thorac Oncol (2009) 4(5):608–14. 10.1097/JTO.0b013e3181a0d98f 19357536

[23] van Beek EJ Mirsadraee S Murchison JT. Lung cancer screening: Computed tomography or chest radiographs? World J Radiol (2015) 7(8):189–93. 10.4329/wjr.v7.i8.189 PMC4553249 26339461

[24] Sozzi G Boeri M Rossi M Verri C Suatoni P Bravi F. Clinical utility of a plasma-based miRNA signature classifier within computed tomography lung cancer screening: a correlative MILD trial study. J Clin Oncol (2014) 32(8):768–73. 10.1200/JCO.2013.50.4357 PMC4876348 24419137

[25] Boeri M Verri C Conte D Roz L Modena P Facchinetti F. MicroRNA signatures in tissues and plasma predict development and prognosis of computed tomography detected lung cancer. Proc Natl Acad Sci USA (2011) 108(9):3713–8. 10.1073/pnas.1100048108 PMC3048155 21300873

[26] Sestini S Boeri M Marchiano A Pelosi G Galeone C Verri C. Circulating microRNA signature as liquid-biopsy to monitor lung cancer in low-dose computed tomography screening. Oncotarget (2015) 6(32):32868–77. 10.18632/oncotarget.5210 PMC4741735 26451608

[27] Perez-Callejo D Romero A Provencio M Torrente M. Liquid biopsy based biomarkers in non-small cell lung cancer for diagnosis and treatment monitoring. Transl Lung Cancer Res (2016) 5(5):455–65. 10.21037/tlcr.2016.10.07 PMC5099509 27826527

[28] Rolfo C Mack PC Scagliotti GV Baas P Barlesi F Bivona TG. Liquid Biopsy for Advanced Non-Small Cell Lung Cancer (NSCLC): A Statement Paper from the IASLC. J Thorac Oncol (2018) 13(9):1248–68. 10.1016/j.jtho.2018.05.030 29885479

[29] Jia Z Patra A Kutty VK Venkatesan T. Critical Review of Volatile Organic Compound Analysis in Breath and In Vitro Cell Culture for Detection of Lung Cancer. Metabolites (2019) 9(3):52. 10.3390/metabo9030052 PMC6468373 30889835

[30] Fiala C Diamandis EP. Utility of circulating tumor DNA in cancer diagnostics with emphasis on early detection. BMC Med (2018) 16(1):166. 10.1186/s12916-018-1157-9 30285732 PMC6167864

[31] Li YL Liu XM Zhang CY Zhou JB Shao Y Liang C. MicroRNA-34a/EGFR axis plays pivotal roles in lung tumorigenesis. Oncogenesis (2017) 6(8):e372. 10.1038/oncsis.2017.50 28825720 PMC5608916

[32] Meng F Henson R Wehbe-Janek H Ghoshal K Jacob ST Patel T. MicroRNA-21 regulates expression of the PTEN tumor suppressor gene in human hepatocellular cancer. Gastroenterology (2007) 133(2):647–58. 10.1053/j.gastro.2007.05.022 PMC4285346 17681183

[33] Feng Z Zhang C Wu R Hu W. Tumor suppressor p53 meets microRNAs. J Mol Cell Biol (2011) 3(1):44–50. 10.1093/jmcb/mjq040 21278451 PMC3030969

[34] Tao J Zhao X Tao J. c-MYC-miRNA circuitry: a central regulator of aggressive B-cell malignancies. Cell Cycle (2014) 13(2):191–8. 10.4161/cc.27646 PMC3906236 24394940

[35] Masliah-Planchon J Garinet S Pasmant E. RAS-MAPK pathway epigenetic activation in cancer: miRNAs in action. Oncotarget (2016) 7(25):38892–907. 10.18632/oncotarget.6476 PMC5122439 26646588

[36] Izzotti A Calin GA Arrigo P Steele VE Croce CM De Flora S. Downregulation of microRNA expression in the lungs of rats exposed to cigarette smoke. FASEB J (2009) 23(3):806–12. 10.1096/fj.08-121384 PMC2653990 18952709

[37] Edmonds MD Eischen CM. Differences in miRNA expression in early stage lung adenocarcinomas that did and did not relapse. PloS One (2014) 9(7):e101802. 10.1371/journal.pone.0101802 25028925 PMC4100742

[38] Gyoba J Shan S Roa W Bedard EL. Diagnosing Lung Cancers through Examination of Micro-RNA Biomarkers in Blood, Plasma, Serum and Sputum: A Review and Summary of Current Literature. Int J Mol Sci (2016) 17(4):494. 10.3390/ijms17040494 27043555 PMC4848950

[39] Yokoi A Matsuzaki J Yamamoto Y Yoneoka Y Takahashi K Shimizu H. Integrated extracellular microRNA profiling for ovarian cancer screening. Nat Commun (2018) 9(1):4319. 10.1038/s41467-018-06434-4 30333487 PMC6192980

[40] Christoph. miRview® lung – Final Approval for Rosetta Genomics’ microRNA Diagnostic Allows Identification of Four Major Subtypes of Lung Cancer, in: miRNA Blog (2012). Available at: https://mirnablog.com/mirview-lung-final-approval-for-rosetta-genomics-microrna-diagnostic-allows-identification-of-four-major-subtypes-of-lung-cancer/ [Accessed 14 March, 2021].

[41] Redis RS Calin S Yang Y You MJ Calin GA. Cell-to-cell miRNA transfer: from body homeostasis to therapy. Pharmacol Ther (2012) 136(2):169–74. 10.1016/j.pharmthera.2012.08.003 PMC3855335 22903157

[42] Arroyo JD Chevillet JR Kroh EM Ruf IK Pritchard CC Gibson DF. Argonaute2 complexes carry a population of circulating microRNAs independent of vesicles in human plasma. Proc Natl Acad Sci USA (2011) 108(12):5003–8. 10.1073/pnas.1019055108 PMC3064324 21383194

[43] Yanaihara N Caplen N Bowman E Seike M Kumamoto K Yi M. Unique microRNA molecular profiles in lung cancer diagnosis and prognosis. Cancer Cell (2006) 9(3):189–98. 10.1016/j.ccr.2006.01.025 16530703

[44] Moretti F D’Antona P Finardi E Barbetta M Dominioni L Poli A. Systematic review and critique of circulating miRNAs as biomarkers of stage I-II non-small cell lung cancer. Oncotarget (2017) 8(55):94980–96. 10.18632/oncotarget.21739 PMC5706930 29212284

[45] Gilad S Lithwick-Yanai G Barshack I Benjamin S Krivitsky I Edmonston TB. Classification of the four main types of lung cancer using a microRNA-based diagnostic assay. J Mol Diagn (2012) 14(5):510–7. 10.1016/j.jmoldx.2012.03.004 22749746

[46] Turchinovich A Samatov TR Tonevitsky AG Burwinkel B. Circulating miRNAs: cell-cell communication function? Front Genet (2013) 4(119):119. 10.3389/fgene.2013.00119 23825476 PMC3695387

[47] Yang M Chen J Su F Yu B Su F Lin L. Microvesicles secreted by macrophages shuttle invasion-potentiating microRNAs into breast cancer cells. Mol Cancer (2011) 10:117. 10.1186/1476-4598-10-117 21939504 PMC3190352

[48] Grange C Tapparo M Collino F Vitillo L Damasco C Deregibus MC. Microvesicles released from human renal cancer stem cells stimulate angiogenesis and formation of lung premetastatic niche. Cancer Res (2011) 71(15):5346–56. 10.1158/0008-5472.CAN-11-0241 21670082

[49] Yu SL Chen HY Chang GC Chen CY Chen HW Singh S. MicroRNA signature predicts survival and relapse in lung cancer. Cancer Cell (2008) 13(1):48–57. 10.1016/j.ccr.2007.12.008 18167339

[50] Kim MK Jung SB Kim JS Roh MS Lee JH Lee EH. Expression of microRNA miR-126 and miR-200c is associated with prognosis in patients with non-small cell lung cancer. Virchows Arch (2014) 465(4):463–71. 10.1007/s00428-014-1640-4 25124149

[51] Yu N Zhang Q Liu Q Yang J. Zhang S. A meta-analysis: microRNAs’ prognostic function in patients with nonsmall cell lung cancer. Cancer Med (2017) 6(9):2098–105. 10.1002/cam4.1158 PMC5603832 28809453

[52] Fondazione IRCCS Istituto Nazionale dei Tumori M. Plasma microRNA Profiling as First Line Screening Test for Lung Cancer Detection: a Prospective Study (BIOMILD). Available at: https://clinicaltrials.gov/ct2/show/NCT02247453 [Accessed 14 March, 2021].

[53] Diagnostics H. Addition of microRNA Blood Test to Lung Cancer Screening Low Dose CT. Available at: https://clinicaltrials.gov/ct2/show/NCT03452514 [Accessed 14 March, 2021].

[54] Humanitas IC. Validation of Multiparametric Models and Circulating and Imaging Biomarkers to Improve Lung Cancer EARLY Detection. (CLEARLY). Available at: https://www.clinicaltrials.gov/ct2/show/NCT04323579 [Accessed 14 March, 2021].

[55] Hospital C-JF. Monitoring the Changes of Tumor-related Biomarkers Before and After Pulmonary Nodule Biopsy. Available at: https://clinicaltrials.gov/ct2/show/NCT03397355 [Accessed 14 March, 2021].

[56] Berard CL. Evaluation of the Feasibility and Clinical Relevance of Liquid Biopsy in Patients With Suspicious Metastatic Lung Cancer (LIBELULE). Available at: https://clinicaltrials.gov/ct2/show/NCT03721120 [Accessed 14 March, 2021].

[57] Sozzi G Boeri M Pastorino U. MS18.02 Circulating Nucleic Acid Biomarkers. J Thoracic Oncol (2019) 14(10):S192–S3. 10.1016/j.jtho.2019.08.383

[58] Wu KL Tsai YM Lien CT Kuo PL Hung AJ. The Roles of MicroRNA in Lung Cancer. Int J Mol Sci (2019) 20(7):1611. 10.3390/ijms20071611 PMC6480472 30935143

[59] Bonneau E Neveu B Kostantin E Tsongalis GJ De Guire V. How close are miRNAs from clinical practice? A perspective on the diagnostic and therapeutic market. EJIFCC (2019) 30(2):114–27. PMC6599191 31263388

[60] Lianidou ES Markou A Strati A. The Role of CTCs as Tumor Biomarkers. In: Advances in Cancer Biomarkers. Dordrecht: Springer (2015). 10.1007/978-94-017-7215-0_21 26530376

[61] Jie XX Zhang XY Xu CJ. Epithelial-to-mesenchymal transition, circulating tumor cells and cancer metastasis: Mechanisms and clinical applications. Oncotarget (2017) 8(46):81558–71. 10.18632/oncotarget.18277 PMC5655309 29113414

[62] Mu Z Benali-Furet N Uzan G Znaty A Ye Z Paolillo C. Detection and Characterization of Circulating Tumor Associated Cells in Metastatic Breast Cancer. Int J Mol Sci (2016) 17(10):1665. 10.3390/ijms17101665 PMC5085698 27706044

[63] Zhu WF Li J Yu LC Wu Y Tang XP Hu YM. Prognostic value of EpCAM/MUC1 mRNA-positive cells in non-small cell lung cancer patients. Tumour Biol (2014) 35(2):1211–9. 10.1007/s13277-013-1162-8 24061641

[64] Chemi F Rothwell DG McGranahan N Gulati S Abbosh C Pearce SP. Pulmonary venous circulating tumor cell dissemination before tumor resection and disease relapse. Nat Med (2019) 25(10):1534–9. 10.1038/s41591-019-0593-1 PMC6986897 31591595

[65] Porras TB Kaur P Ring A Schechter N Lang JE. Challenges in using liquid biopsies for gene expression profiling. Oncotarget (2018) 9(6)7036–53. 10.18632/oncotarget.24140 PMC5805534 29467948

[66] Hannouf MB Zaric GS Blanchette P Brezden-Masley C Paulden M McCabe C. Cost-effectiveness analysis of multigene expression profiling assays to guide adjuvant therapy decisions in women with invasive early-stage breast cancer. Pharmacogenom J (2020) 20(1):27–46. 10.1038/s41397-019-0089-x 31130722

[67] Bracht JWP Gimenez-Capitan A Huang CY Potie N Pedraz-Valdunciel C Warren S. Analysis of extracellular vesicle mRNA derived from plasma using the nCounter platform. Sci Rep (2021) 11(1):3712. 10.1038/s41598-021-83132-0 33580122 PMC7881020

[68] Punnoose EA Atwal S Liu W Raja R Fine BM Hughes BG. Evaluation of circulating tumor cells and circulating tumor DNA in non-small cell lung cancer: association with clinical endpoints in a phase II clinical trial of pertuzumab and erlotinib. Clin Cancer Res (2012) 18(8):2391–401. 10.1158/1078-0432.CCR-11-3148 22492982

[69] Pailler E Adam J Barthelemy A Oulhen M Auger N Valent A. Detection of circulating tumor cells harboring a unique ALK rearrangement in ALK-positive non-small-cell lung cancer. J Clin Oncol (2013) 31(18):2273–81. 10.1200/JCO.2012.44.5932 23669222

[70] Ignatiadis M Litiere S Rothe F Riethdorf S Proudhon C Fehm T. Trastuzumab versus observation for HER2 nonamplified early breast cancer with circulating tumor cells (EORTC 90091-10093, BIG 1-12, Treat CTC): a randomized phase II trial. Ann Oncol (2018) 29(8):1777–83. 10.1093/annonc/mdy211 29893791

[71] CellSearch. THE GOLD STANDARD, in: The first and only actionable test for detecting CTCs in cancer patients. Available at: http://www.cellsearchctc.com/ [Accessed 14 March, 2021].

[72] Lee Y Guan G Bhagat AA. ClearCell(R) FX, a label-free microfluidics technology for enrichment of viable circulating tumor cells. Cytometry Part A J Int Soc Analyt Cytol (2018) 93(12):1251–4. 10.1002/cyto.a.23507 30080307

[73] Sollier-Christen E Renier C Kaplan T Kfir E Crouse SC. VTX-1 Liquid Biopsy System for Fully-Automated and Label-Free Isolation of Circulating Tumor Cells with Automated Enumeration by BioView Platform. Cytometry Part A J Int Soc Analyt Cytol (2018) 93(12):1240–5. 10.1002/cyto.a.23592 PMC6585822 30211979

[74] Miller MC Robinson PS Wagner C O’Shannessy DJ. The Parsortix Cell Separation System-A versatile liquid biopsy platform. Cytometry Part A J Int Soc Analyt Cytol (2018) 93(12):1234–9. 10.1002/cyto.a.23571 PMC6586069 30107082

[75] Hofman V Heeke S Marquette CH Ilie M Hofman P. Circulating Tumor Cell Detection in Lung Cancer: But to What End? Cancers (Basel) (2019) 11(2):262. 10.3390/cancers11020262 PMC6406797 30813420

[76] Kapeleris J Kulasinghe A Warkiani ME Vela I Kenny L O’Byrne K. The Prognostic Role of Circulating Tumor Cells (CTCs) in Lung Cancer. Front Oncol (2018) 8:311. 10.3389/fonc.2018.00311 30155443 PMC6102369

[77] Ilie M Hofman V Long E Bordone O Selva E Washetine K. Current challenges for detection of circulating tumor cells and cell-free circulating nucleic acids, and their characterization in non-small cell lung carcinoma patients. What is the best blood substrate for personalized medicine? Ann Transl Med (2014) 2(11):107. 10.3978/j.issn.2305-5839.2014.08.11 25489581 PMC4245510

[78] Spindler KG Boysen AK Pallisgard N Johansen JS Tabernero J Sorensen MM. Cell-Free DNA in Metastatic Colorectal Cancer: A Systematic Review and Meta-Analysis. Oncol (2017) 22(9):1049–55. 10.1634/theoncologist.2016-0178 PMC5599199 28778958

[79] Fan G Zhang K Ding J Li J. Prognostic value of EGFR and KRAS in circulating tumor DNA in patients with advanced non-small cell lung cancer: a systematic review and meta-analysis. Oncotarget (2017) 8(20):33922–32. 10.18632/oncotarget.15412 PMC5464923 28430611

[80] Han X Wang J Sun Y. Circulating Tumor DNA as Biomarkers for Cancer Detection. Genom Proteomics Bioinf (2017) 15(2):59–72. 10.1016/j.gpb.2016.12.004 PMC5414889 28392479

[81] Schrock AB Welsh A Chung JH Pavlick D Bernicker EH Creelan BC. Hybrid Capture-Based Genomic Profiling of Circulating Tumor DNA from Patients with Advanced Non-Small Cell Lung Cancer. J Thorac Oncol (2019) 14(2):255–64. 10.1016/j.jtho.2018.10.008 30368012

[82] Malapelle U Sirera R Jantus-Lewintre E Reclusa P Calabuig-Farinas S Blasco A. Profile of the Roche cobas(R) EGFR mutation test v2 for non-small cell lung cancer. Expert Rev Mol Diagn (2017) 17(3):209–15. 10.1080/14737159.2017.1288568 28129709

[83] Mok TS Wu YL Ahn MJ Garassino MC Kim HR Ramalingam SS. Osimertinib or Platinum-Pemetrexed in EGFR T790M-Positive Lung Cancer. N Engl J Med (2017) 376(7):629–40. 10.1056/NEJMoa1612674 PMC6762027 27959700

[84] Jenkins S Yang JC Ramalingam SS Yu K Patel S Weston S. Plasma ctDNA Analysis for Detection of the EGFR T790M Mutation in Patients with Advanced Non-Small Cell Lung Cancer. J Thorac Oncol (2017) 12(7):1061–70. 10.1016/j.jtho.2017.04.003 28428148

[85] Gray JE Okamoto I Sriuranpong V Vansteenkiste J Imamura F Lee JS. Tissue and Plasma EGFR Mutation Analysis in the FLAURA Trial: Osimertinib versus Comparator EGFR Tyrosine Kinase Inhibitor as First-Line Treatment in Patients with EGFR-Mutated Advanced Non-Small Cell Lung Cancer. Clin Cancer Res (2019) 25(22):6644–52. 10.1158/1078-0432.CCR-19-1126 PMC7209579 31439584

[86] Douillard JY Ostoros G Cobo M Ciuleanu T McCormack R Webster A. First-line gefitinib in Caucasian EGFR mutation-positive NSCLC patients: a phase-IV, open-label, single-arm study. Br J Cancer (2014) 110(1):55–62. 10.1038/bjc.2013.721 24263064 PMC3887309

[87] Abbosh C Birkbak NJ Wilson GA Jamal-Hanjani M Constantin T Salari R. Phylogenetic ctDNA analysis depicts early-stage lung cancer evolution. Nature (2017) 545(7655):446–51. 10.1038/nature22364 PMC5812436 28445469

[88] Newman AM Bratman SV To J Wynne JF Eclov NC Modlin LA. An ultrasensitive method for quantitating circulating tumor DNA with broad patient coverage. Nat Med (2014) 20(5):548–54. 10.1038/nm.3519 PMC4016134 24705333

[89] Chabon JJ Hamilton EG Kurtz DM Esfahani MS Moding EJ Stehr H. Integrating genomic features for non-invasive early lung cancer detection. Nature (2020) 580(7802):245–51. 10.1038/s41586-020-2140-0 PMC8230734 32269342

[90] Chen M Zhao H. Next-generation sequencing in liquid biopsy: cancer screening and early detection. Hum Genomics (2019) 13(1):34. 10.1186/s40246-019-0220-8 31370908 PMC6669976

[91] Hao X Luo H Krawczyk M Wei W Wang W Wang J. DNA methylation markers for diagnosis and prognosis of common cancers. Proc Natl Acad Sci USA (2017) 114(28):7414–9. 10.1073/pnas.1703577114 PMC5514741 28652331

[92] Kang G Chen K Yang F Chuai S Zhao H Zhang K. and its aberrant methylation in the surveillance of surgical lung Cancer patients: protocol for a prospective observational study. BMC Cancer (2019) 19(1):579. 10.1186/s12885-019-5751-9 31195991 PMC6567563

[93] Ha TT Huy NT Murao LA Lan NT Thuy TT Tuan HM. Elevated levels of cell-free circulating DNA in patients with acute dengue virus infection. PloS One (2011) 6(10):e25969. 10.1371/journal.pone.0025969 22016795 PMC3189230

[94] Cohen JD Li L Wang Y Thoburn C Afsari B Danilova L. Detection and localization of surgically resectable cancers with a multi-analyte blood test. Science (2018) 359(6378):926–30. 10.1126/science.aar3247 PMC6080308 29348365

[95] GRAIL. SUMMIT Study. Available at: https://grail.com/clinical-studies/summit-study/ [Accessed 14 March, 2021].

[96] GRAIL. PATHFINDER Study. Available at: https://grail.com/clinical-studies/pathfinder-study/ [Accessed 14 March, 2021].

[97] GRAIL. Circulating Cell-free Genome Atlas Study. Available at: https://grail.com/clinical-studies/circulating-cell-free-genome-atlas-study/ [Accessed 14 March, 2021].

[98] GRAIL. STRIVE study . Available at: https://grail.com/clinical-studies/strive-study/ [Accessed 14 March, 2021].

[99] Slack FJ Chinnaiyan AM. The Role of Non-coding RNAs in Oncology. Cell (2019) 179(5):1033–55. 10.1016/j.cell.2019.10.017 PMC7347159 31730848

[100] Balatti V Nigita G Veneziano D Drusco A Stein GS Messier TL. tsRNA signatures in cancer. Proc Natl Acad Sci USA (2017) 114(30):8071–6. 10.1073/pnas.1706908114 PMC5544330 28696308

[101] Zhu L Li J Gong Y Wu Q Tan S Sun D. Exosomal tRNA-derived small RNA as a promising biomarker for cancer diagnosis. Mol Cancer (2019) 18(1):74. 10.1186/s12943-019-1000-8 30940133 PMC6444574

[102] Yu Y Xiao J Hann SS. The emerging roles of PIWI-interacting RNA in human cancers. Cancer Manag Res (2019) 11:5895–909. 10.2147/CMAR.S209300 PMC6612017 31303794

[103] Liu Y Dou M Song X Dong Y Liu S Liu H. The emerging role of the piRNA/piwi complex in cancer. Mol Cancer (2019) 18(1):123. 10.1186/s12943-019-1052-9 31399034 PMC6688334

[104] Mei Y Wang Y Kumari P Shetty AC Clark D Gable T. A piRNA-like small RNA interacts with and modulates p-ERM proteins in human somatic cells. Nat Commun (2015) 6(1):7316. 10.1038/ncomms8316 26095918 PMC4557300

[105] Martinez VD Vucic EA Thu KL Hubaux R Enfield KS Pikor LA. Unique somatic and malignant expression patterns implicate PIWI-interacting RNAs in cancer-type specific biology. Sci Rep (2015) 5(1):10423. 10.1038/srep10423 26013764 PMC4444957

[106] Weng W Liu N Toiyama Y Kusunoki M Nagasaka T Fujiwara T. Novel evidence for a PIWI-interacting RNA (piRNA) as an oncogenic mediator of disease progression, and a potential prognostic biomarker in colorectal cancer. Mol Cancer (2018) 17(1):16. 10.1186/s12943-018-0767-3 29382334 PMC5791351

[107] Bhan A Soleimani M Mandal SS. Long Noncoding RNA and Cancer: A New Paradigm. Cancer Res (2017) 77(15):3965–81. 10.1158/0008-5472.CAN-16-2634 PMC8330958 28701486

[108] Huang JL Zheng L Hu YW Wang Q. Characteristics of long non-coding RNA and its relation to hepatocellular carcinoma. Carcinogenesis (2014) 35(3):507–14. 10.1093/carcin/bgt405 24296588

[109] Lin D Watahiki A Bayani J Zhang F Liu L Ling V. ASAP1, a gene at 8q24, is associated with prostate cancer metastasis. Cancer Res (2008) 68(11):4352–9. 10.1158/0008-5472.CAN-07-5237 18519696

[110] Tang R Jiang M Liang L Xiong D Dang Y Chen G. Long Noncoding RNA MALAT-1 Can Predict Poor Prognosis: A Meta-Analysis. Med Sci Monit (2016) 22:302–9. 10.12659/msm.895171 PMC4737057 26821178

[111] Karreth FA Reschke M Ruocco A Ng C Chapuy B Leopold V. The BRAF pseudogene functions as a competitive endogenous RNA and induces lymphoma in vivo. Cell (2015) 161(2):319–32. 10.1016/j.cell.2015.02.043 PMC6922011 25843629

[112] Kalyana-Sundaram S Kumar-Sinha C Shankar S Robinson DR Wu YM Cao X. Expressed pseudogenes in the transcriptional landscape of human cancers. Cell (2012) 149(7):1622–34. 10.1016/j.cell.2012.04.041 PMC3597446 22726445

[113] Poliseno L Marranci A Pandolfi PP. Pseudogenes in Human Cancer. Front Med (Lausanne) (2015) 2(68):68. 10.3389/fmed.2015.00068 26442270 PMC4585173

[114] Han L Yuan Y Zheng S Yang Y Li J Edgerton ME. The Pan-Cancer analysis of pseudogene expression reveals biologically and clinically relevant tumour subtypes. Nat Commun (2014) 5:3963. 10.1038/ncomms4963 24999802 PMC4339277

[115] Hsiao KY Sun HS Tsai SJ. Circular RNA - New member of noncoding RNA with novel functions. Exp Biol Med (Maywood) (2017) 242(11):1136–41. 10.1177/1535370217708978 PMC5478007 28485684

[116] Hansen TB Jensen TI Clausen BH Bramsen JB Finsen B Damgaard CK. Natural RNA circles function as efficient microRNA sponges. Nature (2013) 495(7441):384–8. 10.1038/nature11993 23446346

[117] Li F Zhang L Li W Deng J Zheng J An M. Circular RNA ITCH has inhibitory effect on ESCC by suppressing the Wnt/beta-catenin pathway. Oncotarget (2015) 6(8):6001–13. 10.18632/oncotarget.3469 PMC4467417 25749389

[118] Meng S Zhou H Feng Z Xu Z Tang Y Li P. CircRNA: functions and properties of a novel potential biomarker for cancer. Mol Cancer (2017) 16(1):94. 10.1186/s12943-017-0663-2 28535767 PMC5440908

[119] Barta JA Powell CA Wisnivesky JP. Global Epidemiology of Lung Cancer. Ann Glob Health (2019) 85(1):8. 10.5334/aogh.2419 30741509 PMC6724220

[120] Blandin Knight S Crosbie PA Balata H Chudziak J Hussell T Dive C. Progress and prospects of early detection in lung cancer. Open Biol (2017) 7(9):170070. 10.1098/rsob.170070 28878044 PMC5627048

